# Neural signatures of emotion regulation

**DOI:** 10.1038/s41598-024-52203-3

**Published:** 2024-01-20

**Authors:** Jared Rieck, Julia Wrobel, Antonio R. Porras, Kateri McRae, Joshua L. Gowin

**Affiliations:** 1 Department of Biostatistics and Informatics, University of Colorado Anschutz Medical Campus, Aurora, CO USA; 2 Department of Biostatistics and Bioinformatics, Emory University, Atlanta, GA USA; 3 Department of Pediatrics, Surgery, and Biomedical Informatics, University of Colorado Anschutz Medical Campus, Aurora, CO USA; 4 Department of Pediatric Plastic and Reconstructive Surgery, Children’s Hospital Colorado, Aurora, CO USA; 5 Deparment of Pediatric Neurosurgery, Children’s Hospital Colorado, Aurora, CO USA; 6 Department of Psychology, University of Denver, Denver, CO USA; 7 Department of Radiology, University of Colorado Anschutz Medical Campus, Aurora, CO USA

**Keywords:** Neuroscience, Emotion, Insula, Biomarkers, Predictive markers

## Abstract

Emotional experience is central to a fulfilling life. Although exposure to negative experiences is inevitable, an individual’s emotion regulation response may buffer against psychopathology. Identification of neural activation patterns associated with emotion regulation via an fMRI task is a promising and non-invasive means of furthering our understanding of the how the brain engages with negative experiences. Prior work has applied multivariate pattern analysis to identify signatures of response to negative emotion-inducing images; we adapt these techniques to establish novel neural signatures associated with conscious efforts to modulate emotional response. We model voxel-level activation via LASSO principal components regression and linear discriminant analysis to predict if a subject was engaged in emotion regulation and to identify brain regions which define this emotion regulation signature. We train our models using 82 participants and evaluate them on a holdout sample of 40 participants, demonstrating an accuracy up to 82.5% across three classes. Our results suggest that emotion regulation produces a unique signature that is differentiable from passive viewing of negative and neutral imagery.

## Introduction

Emotion regulation—the ability to change our experience or expression of emotions—is a fundamental human process and has a profound impact on mental health^1^. For example, the strategies that individuals tend to use for emotion regulation can reduce the likelihood of psychopathology when effective, or predispose individuals to developing depression, anxiety, or substance use disorders when they go awry^2,3^. One common emotion regulation strategy, cognitive reappraisal, is defined as “changing how one thinks about a situation to influence one’s emotional response”^4^. Strengthening this skill is the goal of many empirically supported therapeutic interventions, and it forms the basis of cognitive behavioral therapy^5–7^.

While emotion regulation is a clinical target that has been studied experimentally, there remains a need to better understand its biological basis. Numerous neuroimaging studies in humans have explored the circuitry that contributes to emotional response to negative imagery^8,9^ and circuitry involved in modifying a natural response using cognitive reappraisal^10–12^. Empirical work has supported theoretical neural models of emotion^13^, showing that regions such as the amygdala^14^, insula^15^, and striatum^16^ encode salience and negative emotional reactions. The activity of these regions can then be modified by higher-order processes initiated in the anterior cingulate, dorsomedial, dorsolateral, and ventrolateral prefrontal cortex, as well as parietal cortices^17–19^. Negative emotional response may be elevated in adults with mental health problems such as depression^20^, and the higher order influence of emotion regulation may be diminished in adults with psychopathologies^21^. Therefore, understanding the biological bases of both negative emotional response and our ability to control it using emotion regulation is relevant to understanding mental health.

Despite the progress in understanding emotion regulation, much remains unclear. Like many broad neural processes, such as craving^22^ and pain^23^, the neural representation of emotion spans multiple regions and cannot be summarized by the activation of a single structure. A neural signature for picture-induced negative emotion shows that many brain regions contribute to the expression of negative emotion and in aggregate they are highly sensitive and specific to reflecting negative emotion^24^. Thus, our goal is to identify a neural signature that indicates when a person is actively engaging in cognitive processes to modulate their negative emotional response to pictures, which we refer to as reappraisal.

A signature of reappraisal could serve as a biomarker for diagnosis of mental health disorders. It could also serve as a therapeutic target for interventions and one metric of treatment success. Most reappraisal tasks rely upon self-reported negative emotion as their primary measurement, but self-reported emotion is vulnerable to lack of self-awareness of one’s emotions, deliberate deception, and lack of shared linguistic representations of emotion. Therefore, access to a neuroimaging measure as a critical methodological tool that is not subject to the same weaknesses represents a potential methodological advance^25,26^. Although a model has been developed in a sample with mood disorder^27^, to date a signature in a neurotypical population has not been developed, and one study was unsuccessful in developing a signature^28^.

Here, we use novel approaches to develop a signature of reappraisal in data from 82 healthy young adults. In developing this signature, we compare the results of two machine learning models: the commonly employed LASSO principal component regression (LASSO PCR) and linear discriminant analysis (LDA). Specifically, we train the models to classify images into three categories defined by an fMRI task and instruction: neutral imagery with an instruction to look at the image (neutral), negative imagery with an instruction to look at the image (negative), and negative imagery with an instruction to regulate the emotional response to feel less negatively (decrease). The LASSO PCR modeling approach largely mirrors analyses from previous studies^24,29^, with adaptation to accommodate the multi-class nature of the data. The fundamentally different LDA procedure is a supervised machine learning model that utilizes linear discriminants as predictors in a multinomial logistic regression classifier without LASSO regularization. The predictive performance of these models was evaluated both via cross-validation and in a holdout dataset of 40 adults collected at a different site. Employing both models allows us to establish which method can better predict the emotion regulation task, and to compare regions that may drive this prediction.

## Results

### Predictive performance

**Figure 1:**
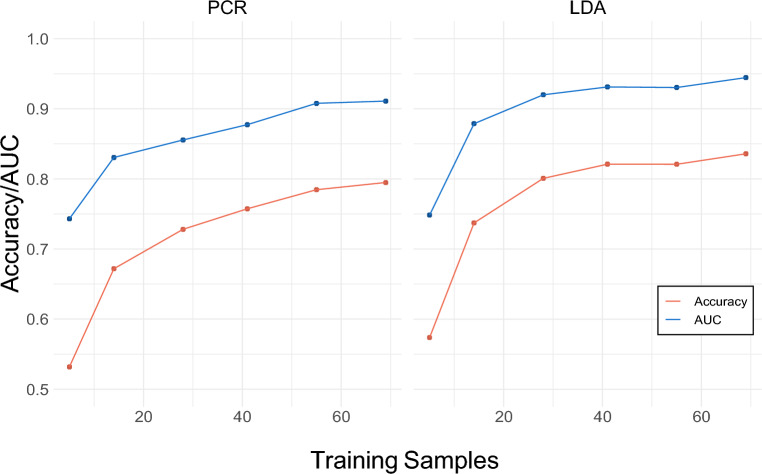
Model cross-validated accuracy and AUC. Results from cross-validation are presented averaged over 20 models trained on each of six training sample sizes. The left panel displays accuracy and AUC (area under the receiver operating characteristic curve) for the LASSO PCR model while the right panel displays accuracy and AUC for the LDA model.

Figure 1 shows AUC and accuracy on six training sample sizes of the cross-validated training data, intended to show the changes in modeling metrics as more training samples are provided. Assessing accuracy and AUC in cross-validation also provides evidence of the extent to which the models are overfitting to training data. The LASSO PCR model achieved an average cross- validated accuracy of 0.791 for models trained on 84% of the sample (n = 69) with a corresponding AUC of 0.912. For both models, accuracy and AUC both increase as the number of training samples is increased. The LDA model slightly outperformed the LASSO PCR model at all training sample sizes in terms of both accuracy and AUC. For LDA models trained on 84% of the sample (n = 69), average accuracy was 0.836 with a corresponding AUC of 0.944. Figure 1 also indicates that adding participants beyond 69 would likely produce only small increases in performance.

**Figure 2:**
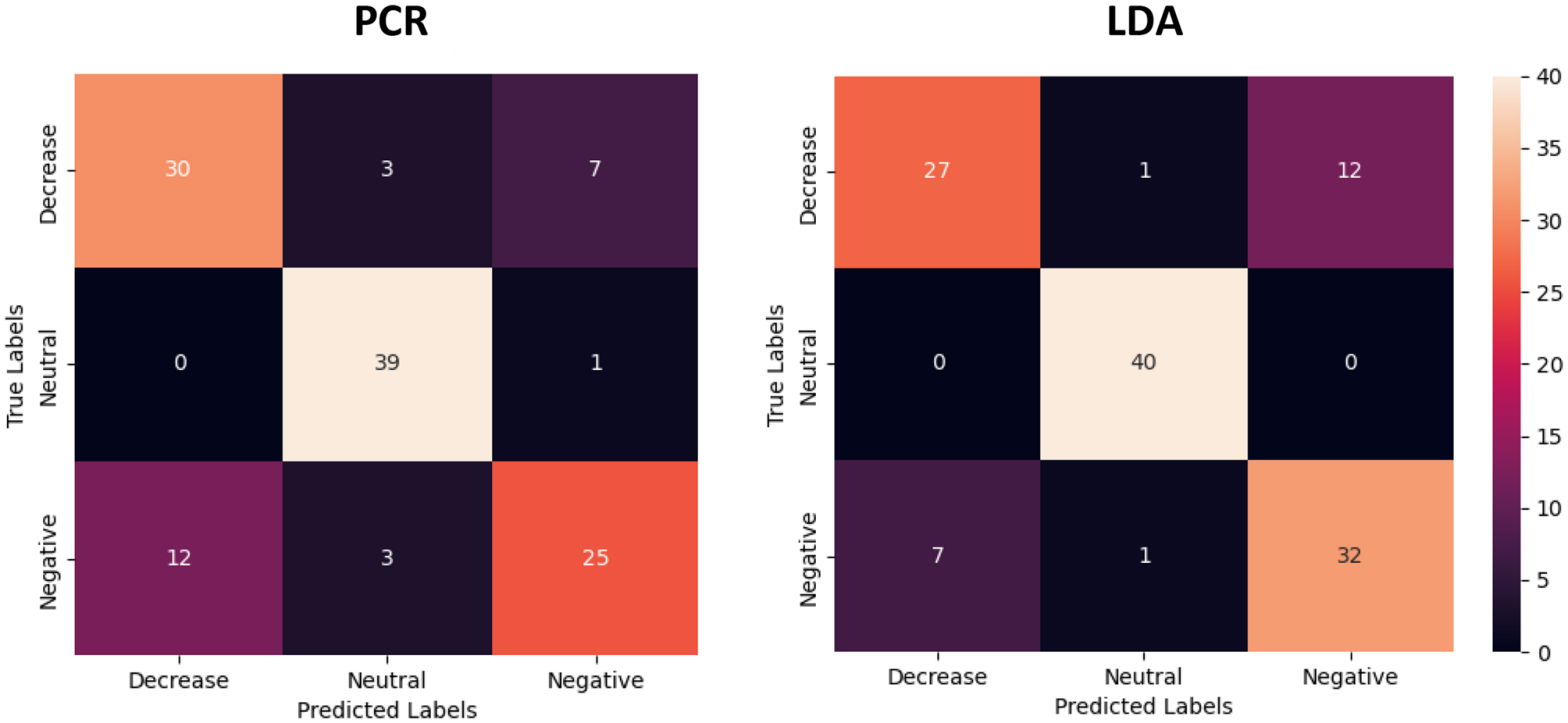
Holdout confusion matrices. LASSO PCR and LDA classification performances in the holdout data. Diagonals represent correctly classified samples while off diagonals contain the misclassified samples.

Figure 2 displays confusion matrices of LASSO PCR and LDA classification performance in the holdout data. In the holdout data, the LASSO PCR model achieved an overall accuracy of 78.3% with a corresponding AUC of 0.916 (Table 1). Class-wise sensitivity was highest for the neutral class (0.975) and lowest for the negative class (0.625). The LDA model achieved an overall accuracy of 82.5% in the holdout data with a corresponding AUC of 0.944. No neutral class samples were misclassified, whereas class-wise sensitivity was lowest for decrease (0.675). While accuracy and AUC in the holdout data were higher for the LDA model, differences in classification performance these models in the holdout data were not statistically significant (p = 0.424).

**Table 1:** Holdout modeling metrics.

Class	LASSO PCR				LDA			
	Accuracy	AUC	Sensitivity	Specificity	Accuracy	AUC	Sensitivity	Specificity
Decrease		0.901	0.750	0.842		0.916	0.675	0.911
Negative		0.866	0.625	0.896		0.915	0.800	0.848
Neutral		0.995	0.975	0.902		1.000	1.000	0.967
Overall	0.783	0.916	0.783	0.880	0.825	0.944	0.825	0.909

**Figure 3:**
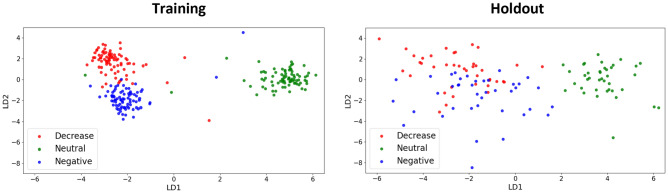
LDA class separation. The distribution of training samples in the reduced two-dimensional feature space produced by LDA (left panel) and the distribution of CovBat harmonized holdout samples after projection of LD loadings from training (right panel).

Figure 3 visualizes class separation for the LDA model in the training and holdout data sets. The distribution of training samples in the reduced two-dimensional feature space produced by LDA produces clear class separation, with the decrease and negative classes separated from the neutral class along the LD1 axis, while decrease is separated from negative along the LD2 axis. In order to mitigate site effects which impact the scale of linear transformations of the neural activation maps, we performed CovBat harmonization on the holdout data (Supplementary Figs. S1, S2). The same pattern of class separation was observed in the holdout data, with higher intraclass variance and a greater degree of overlap between the decrease and negative classes.

### Identifying the emotion regulation signature

**Figure 4:**
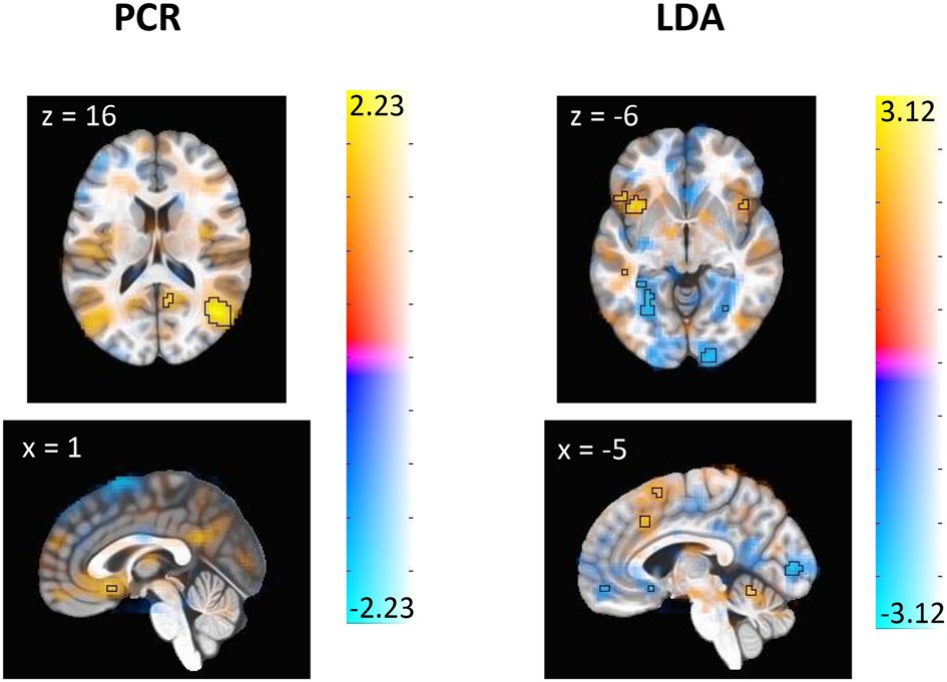
Thresholded display of emotion regulation signature. These images display brain regions which contribute most consistently to the identification of emotion regulation. Color shows the average contribution of each voxel to the prediction of emotion regulation, with warm color regions depicting positive associations and cool color regions depicting negative associations. Outlined regions display the voxels whose contributions are significant at the adjusted p < 0.005 threshold for LASSO PCR (left panel) and LDA (right panel). Note that slice location is not uniform between panels.

Figure 4 shows the emotion regulation signature from the LASSO PCR and LDA models, obtained via bootstrap. The voxels that are predictive of the modulation task based on the LASSO PCR model occur primarily in occipital lobe and ventrolateral PFC, while the predictive voxels derived from the LDA model occur in the insula, dorsomedial PFC, anterior lobe of cerebellum, occipital lobe, cingulate gyrus, orbitofrontal PFC, and ventrolateral PFC. Tables 2 and 3 complement Fig. 4 by highlighting the brain regions of specific clusters of voxels that significantly drive class prediction for each model. These clusters of emotion regulation signature weights reveal that relatively fewer brain regions are identified as being predictively important of the emotion regulation task by the LASSO PCR model as compared to the LDA model. Both models produce clusters of weights in Brodmann Areas 6 and 40 and additionally identify prefrontal cortex as a predictively important region in the emotion regulation task.

**Table 2:** Clusters of bootstrapped emotion regulation signature weights—PCR.

Volume (mm^3^)	x	y	z	Region	Brodmann Area	Max(z)
Positive weights						
3321	46	− 65	17	Right temporoparietal Junction	BA 39	2.22
891	− 47	− 32	59	Left postcentral gyrus	BA 40	1.90
648	24	− 27	− 4	Right hippocampus	BA 27	1.88
Negative weights						
3510	− 52	− 57	43	Left inferior parietal lobule	BA 40	− 2.01
1107	− 44	7	52	Left precentral gyrus	BA 6	− 1.99
675	− 5	10	67	Left superior frontal gyrus	BA 6	− 1.86

**Table 3:** Clusters of bootstrapped emotion regulation signature weights—LDA.

Volume (mm^3^)	x	y	z	Region	Brodmann area	Max(z)
Positive weights						
1890	− 49	− 61	47	Left inferior parietal lobule	BA 40	2.72
1782	− 40	19	− 1	Left anterior insula	BA 47	2.91
1296	− 53	− 37	3	Left middle temporal gyrus	BA 22	2.78
702	− 10	13	62	Left superior frontal gyrus	BA 6	2.52
540	42	− 70	− 13	Right fusiform gyrus	BA 19	2.49
513	45	− 49	− 22	Right fusiform gyrus	BA 37	2.77
432	− 6	− 60	− 15	Cerebellar vermis		2.82
432	41	− 30	52	Right postcentral gyrus	BA 3	2.26
Negative weights						
1944	31	− 83	23	Right middle occipital gyrus	BA 19	− 2.63
1836	− 30	− 43	− 11	Left fusiform gyrus	BA 37	− 3.13
1809	15	− 51	17	Right precuneus	BA 30	− 3.04
1755	− 13	− 55	15	Left precuneus	BA 30	− 2.83
1755	− 41	− 23	51	Left postcentral gyrus	BA 3	− 2.54
1512	− 49	− 23	15	Left supramarginal gyrus	BA 40	− 2.92
1026	− 34	− 84	28	Left middle occipital gyrus	BA 19	− 2.50
999	− 8	− 92	5	Left calcarine gyrus	BA 17	− 2.50
999	13	− 40	48	Right middle cingulate cortex	BA 7	− 2.72
945	29	− 39	− 12	Right fusiform gyrus	BA 37	− 2.67
540	17	− 98	− 7	Right lingual gyrus	BA 17	− 2.43
513	49	− 33	30	Right supramarginal gyrus	BA 40	− 2.48
405	2	16	− 10	Left subgenual cingulate cortex	BA 25	− 2.73

### Self-reported scores of negative emotion

In the training sample, self-reports of negative affect were highest for the negative class (mean = 3.502, sd = 0.655) with lower reported scores for decrease (mean = 2.811, sd = 0.605) and neu- tral (mean = 1.111, sd = 0.196). A total of 14 subjects (17.1%) reported negative affect during the decrease condition as compared to passive viewing of negative imagery. In the holdout sample, similar trends are observed with the highest self-reported scores of negativity observed for the negative class (mean = 3.943, sd = 0.926) with lower scores for decrease (mean = 3.338, sd = 0.926) and neutral (mean = 1.141, sd = 0.315). In this sample, 11 subjects (27.5%) reported higher negative affect during the emotion regulation task as compared to passive viewing of negative imagery.

## Discussion

In this study, we explored the neural signatures of emotion regulation among a population of neurotypical young adults using two machine learning methods. Both methods produced accurate predictions of the fMRI task when evaluated in cross-validation and in a holdout dataset. This accuracy is noteworthy because the external stimuli are highly similar for passive viewing versus active reappraisal of negative imagery; the only difference is the internal thinking associated with cognitively changing the meaning of the negative stimuli. The predictive performance of these models indicates that despite the similarity of these stimuli, it is possible to determine when an individual engages in cognitive reappraisal using neural activation patterns.

Our signatures suggest that engagement in emotion regulation can be detected based on activation levels from brain regions previously shown to be involved in emotion regulation based on meta-analysis^30,31^, in addition to contributions from regions not identified in these reports. For example, meta-analyses indicate that emotion regulation engages regions in the prefrontal cortex (i.e., ventromedial PFC, ventrolateral PFC, dorsomedial PFC), along with insular cortex and left superior frontal gyrus^12,32,33^. We similarly identify these regions as contributors to the signature of emotion regulation, suggesting that our model captures neural processing patterns consistent with existing empirical and theoretical models of emotion regulation^13^. At the same time, these regions are also engaged during distraction tasks^34^, raising the possibility that the signature captures neural activity related to cognitive control in general, rather than reappraisal specifically. The present experimental design (look-negative, look-neutral, decrease-negative) makes it difficult to disentangle these possibilities, so future studies should test whether our signature can discriminate between general cognitive effort versus the specific act of cognitive reappraisal. Unlike previous meta-analyses, our signature identified occipital cortex regions in the signature of emotion regulation, which is similar to the findings from the picture-induced negative emotion signature^24^. While visual processing regions may have a cognitive role in emotion processing, they may also simply be attuned to the salience of visual imagery. This possibility again indicates that to understand the role of visual regions in the neural signature, future experiments are needed with designs that allow researchers to disentangle salience of visual cues from emotion reactivity and regulation. These possibilities also suggest a fundamental limitation of multivariate classifiers, since they may not be able to discriminate between brain regions that are ‘mission critical’ to cognition and those that are merely correlated with the activity. Given the clinical importance of cognitive reappraisal, continued refinement and understanding is useful, even if neural processes specific to cognitive reappraisal are difficult to tease apart from related cognitive activities.

While many previous studies have utilized LASSO PCR to identify brain regions which are associated with performing an fMRI task, we choose to additionally present the signature derived from LDA in order to provide a more comprehensive and robust representation of reappraisal. In doing so, we establish increased confidence in the involvement of brain regions identified by both models. Specifically, prefrontal cortex and left superior frontal gyrus are identified in the signatures produced by both models, validating prior accounts of the involvement of these regions in higher order processes^35^. While the usage of LASSO regularization reduces overfitting, it also produces a reappraisal signature that is sparse relative to LDA, identifying fewer involved brain regions and producing bootstrapped cluster weights with lower total volumes. While most of the information that is discarded by LASSO regularization is likely noise, some signal is likely discarded as well. Consequently, some relevant brain regions may not be identified in the PCR-derived signature. Meanwhile, the signature derived from the LDA model is more susceptible to overfitting in high-dimensional data, which may also contribute to the identification of many more small “meaningful” regions. . It must be considered that the validity of the LASSO PCR modeling approach rests on the assumption that there is a relationship between the variance in the dataset and the fMRI task, an assumption which is not required by the LDA model as it is a supervised method that partitions the data on the basis of a known class separation structure^44^. Although LASSO PCR is a standard method for the development of signatures derived from task-based fMRI data, these aspects suggest that comparing and contrasting results from different methods may help in developing a comprehensive understanding of the underlying associations between brain regions and the fMRI task. While the unique advantages and disadvantages of these models must be kept in mind, it is also worth considering that the slightly stronger classification performance of the LDA model may indicate that the derived signature identifies more reappraisal-specific regions.

This modeling of a neural signature of emotion regulation meets an important criterion for generalizability since it was developed on a dataset from one site and showed similar accuracy for data collected at a different site. To assess performance on holdout data, it was necessary to adjust for site-specific differences in scanners and data pre-processing steps. While prior work has utilized COMBAT harmonization to address site effects in functional connectivity measurements^36^, more recent work has shown that making an additional correction for feature covariance (CovBat) further reduces the impact of site effects on machine learning models applied to neuroimaging data^37^. Applying CovBat by class largely eliminated site-specific distributional differences. The accuracy and AUC achieved by both models in the harmonized holdout dataset is similar to the values achieved in internal cross-validation, suggesting the results generalize to other samples. Notably, both models discriminate the neutral class from decrease and negative with nearly perfect accuracy, while also achieving high classification accuracy of the negative and decrease classes.

One limitation worth considering in this analysis is in the degree to which subjects were successful in performing the task of emotional modulation that is characteristic of the decrease class. That is, while self-reports of negative affect were lower in the decrease class on average as compared to negative, there remains a substantial subset of subjects in both the training and holdout samples who reported feeling more negatively when given the decrease-negative instruction as compared to look-negative, indicating a potential failure to regulate the emotional response. If individuals who report feeling more negatively during the emotion regulation task in fact have distinct signatures from those who report feeling less negatively, this effect could potentially be captured by integrating self-report information as a separate data modality during training. On the other hand, the self-reported changes in negative affect have not been shown to distinguish groups with clinical disorders from healthy samples, whereas neural activation patterns have, so it may be that the neural signature could be useful for clinical populations even if the behavioral results do not align^38^. Another limitation is the exploratory nature of this signature, as we did not test a hypothesis with our analyses. We used an agnostic approach to the brain, with each voxel in the brain being weighted equally in analysis. Although examining regions of concordance from multiple modeling approaches can help reduce the odds of picking up on spurious associations, these results would be strengthened by confirmatory studies. Finally, it is important to note that the derivation of the signature is from a multinomial model, rather than a binary classifier which discriminates between the look-negative and decrease-negative conditions. Correspondingly, identified predictive voxels should be understood as the regions which differentiate reappraisal from passive viewing of both similar (look-negative) and dissimilar (look-neutral) imagery.

This work extends our understanding of the emotional response by exploring the signatures that are unique to conscious control of emotion, while also validating previously published accounts of signatures which are characteristic of negative emotional experience. Our results further show that extensions of the LASSO principal components regression and linear discriminant analysis models to multiclass neuroimaging data produce signatures that are highly accurate at distinguishing emotion regulation tasks both in cross-validation and holdout analyses. This fMRI-based neuromarker serves to further our understanding of the circuitry involved in cognitive reappraisal and may lend itself towards future explorations of psychopathologies and their treatment.

## Methods

Experimental procedures for the primary sample were approved by the Colorado Multiple Institutional Review Board (COMIRB) under protocol 18-1426. All participants provided written informed consent, and all methods were performed in accordance with the relevant guidelines and regulations.

### Participants

The training data consists of eighty-two subjects (mean age = 20.95, SD = 1.34, 54.2% Female, 77.2% Caucasian) recruited from the Denver metro area. Participants were eligible if they were between the ages of 18 and 22 at time of study enrollment and were MRI eligible. Exclusion criteria were (1) being treated for a psychiatric disorder, (2) seeking treatment for alcohol use disorder, (3) regular tobacco use, (4) using medications that affect the hemodynamic response, (5) history of head trauma with loss of consciousness, (6) evidence of cannabis misuse (defined as > 8 uses of cannabis per month or a score > 11 on the Cannabis Use Disorder Identification Test- Revised), (7) > 10 lifetime uses of illicit substances, (8) misuse of prescription medication in the prior year, or (9) pregnancy.

Forty subjects (mean age = 25.5, SD = 4.7, 70% Female, 87.5% Caucasian) were recruited from the Boulder, CO community for a separate study on cognitive reappraisal^39^. Subjects in this sample were given the same set of fMRI tasks, and these forty subjects were used as holdout data to assess the generalizability of the models.

### Task and stimuli

Each subject was shown images from the International Affective Picture System (IAPS), a database containing a standardized set of images adopted widely in psychological research for the study of emotion and attention^40^. Images displayed to subjects in this study consisted of 15 neutral images and 30 negative images. The 15 neutral images were paired with a “look” instruction to the subject, indicating that the participant should simply maintain their attention on the visual stimulus. 15 negative images were paired with the same “look” instruction while the remaining 15 were paired with a “decrease” instruction, indicating that the participant should observe the aversive photo while attempting to consciously modulate their emotional response to feel less negatively. The instruction was shown to the subject for two seconds prior to display of the image for seven seconds. Display of the image was followed by a rest period of between one and three seconds, after which participants were given four seconds to provide a score on how negatively they felt after viewing the image on a scale from 1 (not at all negative) to 5 (very negative).

Prior to the scan, each participant was instructed on what strategy to use for the “decrease” condition. They were specifically asked to “think of something to tell yourself that helps you to feel less negative about the picture. So for example, you could tell yourself something about the outcome, so that whatever is going on will soon be resolved, or that help is on the way. You could also focus on a detail or aspect of the situation that isn’t quite as bad as it first seemed. But we want you to stay focused on the picture and not think of random things that make you feel better, but rather to change something about the picture that helps you to feel less negative about it.” After the instruction, each participant was asked to practice a “decrease” strategy out loud while viewing a negative image so that the research assistant could confirm understanding and correct use of reappraisal.

### MRI acquisition

Images gathered for the training sample were collected on a Siemens 3.0 Tesla Skyra Magnet with a 20-channel head coil. Functional images were acquired using BOLD signal across 40 axial slices with TR = 2000-ms, TE = 30-ms, flip angle = 77, field of view = 220-mm, 40 axial 3-mm thick slices, and multiband slice acceleration factor of 2 to increase spatial resolution while maintaining temporal signal-to-noise ratio. Images were acquired in oblique orientation and high-resolution T1-weighted images were obtained for anatomical reference.

Images gathered for the holdout sample were collect on a 3.0 Tesla Siemens Magnetom Prisma system with a 32-channel head coil, with functional images acquired using BOLD signal across 56 contiguous slices with parameters TR = 460-ms, TE = 27.2-ms, flip angle = 44°. and 3-mm thick slices^30^.

### Data preprocessing

Data preprocessing was performed with Analysis of Functional NeuroImages (AFNI) Version 22.0.21^41^. All images were converted into AFNI-compatible file formats and echoplanar images were aligned with anatomical images. Spatial normalization was performed by nonlinearly warping the anatomical data to the Montreal Neurological Institute (MNI) standard space using AFNI SSwarper^42^. Images were skull-stripped deobliqued using the MNI152_2009_template_SSW.nii.gz template (https://afni.nimh.nih.gov/pub/dist/doc/htmldoc/template_atlas/sswarper_base.html). The final voxel resolution was 3-mm isotropic. Time points with greater than 0.3-mm Euclidean distance of framewise displacement were censored from analyses. An 8-mm kernel was used for blur, and each run was scaled to produce a mean intensity for each voxel of 100. A 7-s block model was applied to regression analysis to obtain voxel-level beta weights for each class of stimulus. Regressors of interest were the presentation of each cue (look-neutral, look-negative, decrease-negative). We included six regressors of no interest to account for motion of translation and rotation in the x, y, and z dimensions. Beta values from this regression model were then utilized as class-specific activation maps (beta maps). Images gathered for holdout analysis followed a separate preprocessing pipeline detailed in Powers et al.^39^.

### LASSO principal components regression

Principal components analysis was performed on the training data, and 65 principal components were retained to reach a threshold of 90% cumulatively explained variance. These principal com- ponents were then utilized as predictors of the stimulus class in a LASSO penalized multinomial logistic regression model, where the LASSO tuning parameter *λ* was selected as the value which minimized multinomial deviance in tenfold cross-validation. When predicting on test data, the principal component loadings from the training data were projected onto the test data.

### Linear discriminant analysis

Linear discriminant analysis is a method of identifying a linear combination of features to separate two or more classes of data in a manner that maximizes the ratio of between-class variance to within-class variance^43^. We utilize LDA in this context to reduce the dimensionality of the imaging features in a manner similar to the usage of PCA in the LASSO PCR model, but with the distinction that LDA is a supervised means of dimension reduction that explicitly considers the differences between the classes of data rather than building a reduced set of features based on feature variance, guaranteeing maximal class separability^44^. LDA using two components was performed on the training data, with loadings projected onto test data for prediction. A multinomial logistic regression model was fit to the training data using the two linear discriminants as predictors. Visualizations of observations of each class were constructed to evaluate the linear separability of the classes.

### Feature harmonization

Given that the training and holdout data were collected at different institutions with distinct pre-processing pipelines and different scanners, it was necessary to harmonize the distributions of features to mitigate site effects. To this end, CovBat^37^ was performed separately on beta maps for each class of stimulus, harmonizing the voxel-level distributions in the training and holdout data. Splitting the data by class prior to harmonization leverages the fact that we anticipate beta weight distributions at many voxels to differ by class, as do we anticipate that each class will have a unique covariance matrix, as is evidenced by site-specific distributional differences in PC distributions by class which are mitigated after harmonization (Supplementary Figs. S1, S2).

### Assessing predictive performance.

Predictive performance was assessed on the training and test sets using measures of accuracy and area under the curve (AUC). On the training data, cross-validation was performed on each of six training sample sizes (5, 14, 28, 41, 55, 69). For cross-validation, multiclass AUC was calculated as described in Hands and Till^45^. The same method was used to calculate overall AUC for both models in the holdout dataset, while the class-wise AUCs reported in Table 1 were calculated using the one-versus-rest approach. Predictive performance of the PCR and LDA models were compared in the harmonized test data using McNemar’s test.

### Voxel-wise contributions to class prediction

We used the bootstrap approach described in Koban et al.^22^ to obtain voxel-level p-values and test the reliability of each voxel’s contribution to class prediction. This strategy was used for both the LASSO PCR and LDA models using the same 1000 bootstrap samples. Specifically, for each bootstrap sample a LASSO PCR and an LDA model were fit. Then, for each bootstrap sample and each class k a vector of weights the same length as the number of voxels in each image was calculated by *W*_*k*_ = *Vβ*_*k*_. Here *W*_*k*_ is a vector of contribution weights for class k and *β*_*k*_ is the vector of fitted multinomial logistic regression coefficients for class k. For LASSO PCA, *V* is a matrix of principle component loadings selected as nonzero by LASSO, and for LDA *V* is a 2-column matrix of linear discriminant values.

The sign of these weights were recorded across the 1000 bootstrap samples, and voxels which made consistently positive or negative contributions to the prediction of a class were identified based on a threshold of p < 0.005, indicating that the voxel contributed either positively or negatively to the prediction of the decrease class at least 99.5% of the time. Voxels meeting this threshold were visualized by thresholding according to the voxel’s z-statistic from the bootstrapped distribution of contribution weights.

### Supplementary Information

Supplementary Figures.

## Data Availability

The data that support these findings are available from the corresponding author J.R. upon reasonable request.

## References

[1] Gross JJ, John OP (2003). Individual differences in two emotion regulation processes: Implications for affect, relationships, and well-being. J. Pers. Soc. Psychol..

[2] Gross JJ, Munoz RF (1995). Emotion regulation and mental health. Clin. Psychol. Sci. Pract..

[3] Stellern J (2022). Emotion regulation in substance use disorders: A systematic review and meta-analysis. Addiction.

[4] McRae K, Gross JJ (2020). Emotion regulation. Emotion.

[5] Lazarus RS, Alfert E (1964). Short-circuiting of threat by experimentally altering cognitive appraisal. J. Abnormal Soc. Psychol..

[6] BECK, A. T. Thinking and depression. *Archives of General Psychiatry* **9**, 324 (1963). 10.1001/archpsyc.1963.01720160014002 14045261

[7] Beck, A. T. *Cognitive therapy of depression*. (Guilford Press, 2001).

[8] Ochsner KN, Bunge SA, Gross JJ, Gabrieli JD (2002). Rethinking feelings: An fmri study of the cognitive regulation of emotion. J. Cogn. Neurosci..

[9] Ball TM (2012). Selective effects of social anxiety, anxiety sensitivity, and negative affectivity on the neural bases of emotional face processing. NeuroImage.

[10] Wager TD, Davidson ML, Hughes BL, Lindquist MA, Ochsner KN (2008). Prefrontal- subcortical pathways mediating successful emotion regulation. Neuron.

[11] Buhle JT (2013). Cognitive reappraisal of emotion: A meta-analysis of human neuroimaging studies. Cereb. Cortex.

[12] Frank DW (2014). Emotion regulation: Quantitative meta-analysis of functional activation and deactivation. Neurosci. Biobehav. Rev..

[13] Ochsner K, Gross J (2005). The cognitive control of emotion. Trends Cogn. Sci..

[14] Costafreda SG, Brammer MJ, David AS, Fu CHY (2008). Predictors of amygdala activation during the processing of emotional stimuli: A meta-analysis of 385 PET and fmri studies. Brain Res. Rev..

[15] Stein MB, Simmons AN, Feinstein JS, Paulus MP (2007). Increased amygdala and insula activation during emotion processing in anxiety-prone subjects. Am. J. Psychiatry.

[16] O’Doherty JP, Deichmann R, Critchley HD, Dolan RJ (2002). Neural responses during anticipation of a primary taste reward. Neuron.

[17] Goldin PR, McRae K, Ramel W, Gross JJ (2008). The neural bases of emotion regulation: Reappraisal and suppression of negative emotion. Biol. Psychiatry.

[18] Morawetz C, Bode S, Derntl B, Heekeren HR (2017). The effect of strategies, goals and stimulus material on the neural mechanisms of emotion regulation: A meta-analysis of fMRI studies. Neurosci. Biobehav. Rev..

[19] Morawetz C, Riedel MC, Salo T, Berboth S, Eickhoff SB, Laird AR, Kohn N (2020). Multiple large-scale neural networks underlying emotion regulation. Neurosci. Biobehav. Rev..

[20] Groenewold NA, Opmeer EM, de Jonge P, Aleman A, Costafreda SG (2013). Emotional valence modulates brain functional abnormalities in depression: Evidence from a meta-analysis of fmri studies. Neurosci. Biobehav. Rev..

[21] Zilverstand A, Parvaz MA, Goldstein RZ (2017). Neuroimaging cognitive reappraisal in clinical populations to define neural targets for enhancing emotion regulation A systematic review. NeuroImage.

[22] Koban L, Wager TD, Kober H (2022). A neuromarker for drug and food craving distinguishes drug users from non-users. Nat. Neurosci..

[23] Wager TD (2013). An fmri-based neurologic signature of physical pain. N. Engl. J. Med..

[24] Chang LJ, Gianaros PJ, Manuck SB, Krishnan A, Wager TD (2015). A sensitive and specific neural signature for picture-induced negative affect. PLOS Biol..

[25] Farah MJ, Hutchinson JB, Phelps EA, Wagner AD (2014). Functional MRI-based lie detection: Scientific and societal challenges. Nat. Rev. Neurosci..

[26] Tang J, LeBel A, Jain S, Huth AG (2023). Semantic reconstruction of continuous language from non-invasive brain recordings. Nat. Neurosci..

[27] Schneck N (2023). The temporal dynamics of emotion regulation in subjects with major depression and healthy control subjects. Biol. Psychiatry.

[28] Zhang, J. X., Dixon, M. L., Goldin, P. R., Spiegel, D., & Gross, J. J. The neural separability of emotion reactivity and regulation. 10.1007/s42761-023-00227-9 PMC10751283 38156247

[29] Coll, M.-P. *et al.* The neural signature of the decision value of future pain. *Proc. Natl. Acad. Sci.* **119**, (2022). 10.1073/pnas.2119931119 PMC9191656 35658082

[30] Buhle JT, Silvers JA, Wager TD, Lopez R, Onyemekwu C, Kober H, Weber J, Ochsner KN (2013). Cognitive reappraisal of emotion: A meta-analysis of human neuroimagingstudies. Cereb. Cortex.

[31] Picó Pérez, M. *Emotion regulation in mood and anxiety disorders: A meta-analysis of fMRI cognitive reappraisal studies* (2017). 10.1016/j.pnpbp.2017.06.001 28579400

[32] Morawetz C (2017). The effect of strategies, goals and stimulus material on the neural mechanisms of emotion regulation: A meta-analysis of fMRI studies. Neurosci. Biobehav. Rev..

[33] Kohn, N., *et al.* Neural network of cognitive emotion regulation—an ALE meta-analysis and MACM analysis. *Neuroimage* **87**, 345–355 (2014). 10.1016/j.neuroimage.2013.11.001 PMC4801480 24220041

[34] McRae, K., *et al.* The neural bases of distraction and reappraisal. *J. Cogn. Neurosci.* **22**(2), 248–262 (2010). 10.1162/jocn.2009.21243 PMC4136451 19400679

[35] Boisgueheneuc F, d., Levy, R., Volle, E., Seassau, M., Duffau, H., Kinkingnehun, S., Samson, Y., Zhang, S., & Dubois, B.  (2006). Functions of the left superior frontal gyrus in humans: A lesion study. Brain.

[36] Yu M (2018). Statistical harmonization corrects site effects in functional connectivity measurements from multi-site fmri data. Hum. Brain Mapp..

[37] Chen AA (2021). Mitigating site effects in covariance for machine learning in neuroimaging data. Hum. Brain Mapp..

[38] Mcrae, Kateri (2023). *Neural Bases of Emotion Regulation*. [Manuscript in-press].

[39] Powers JP, Kako N, McIntosh DN, McRae K (2022). Competitive interactions between cognitive reappraisal and mentalizing. Int. J. Psychophysiol..

[40] Lang, P. J., Bradley, M. M. & Cuthbert, B. N. *International affective picture system (IAPS): Affective ratings of pictures and instruction manual*. (NIMH, Center for the Study of Emotion & Attention, 2005).

[41] Cox RW (1996). AFNI: Software for analysis and visualization of functional magnetic resonance neuroimages. Comput. Biomed. Res..

[42] Kirk-Provencher, K. T., Gowin, J. L., McRae, K. & Penner, A. E. Emotion regulation in young adults with family history of harmful alcohol use: A fmri study. *Drug Alcohol Depend*. 10.1016/j.drugalcdep.2022.109752 PMC9875721 36610254

[43] Balakrishnama S, Ganapathiraju A (1998). Linear discriminant analysis-a brief tutorial. Inst. Signal Inf. Process..

[44] Xanthopoulos, P., Pardalos, P. M. & Trafalis, T. B. Linear discriminant analysis. in *Robust data mining* 27–33 (Springer New York, 2013). 10.1007/978-1-4419-9878-1_4.

[45] Hand DJ, Till RJ (2001). A simple generalisation of the area under the ROC curve for multiple class classification problems. Mach. Learn..

[46] Lindquist KA, Wager TD, Kober H, Bliss-Moreau E, Barrett LF (2012). The brain basis of emotion: A meta-analytic review. Behav. Brain Sci..

[47] Sato JR (2015). Machine learning algorithm accurately detects fmri signature of vulnerability to major depression. Psychiatry Res. Neuroimaging.

