# Supplementary material for: Neural signatures of emotion regulation
Source: Sci Rep. 2024 Jan 20;14:1775. doi: 10.1038/s41598-024-52203-3 (PMC10799868; doi:10.1038/s41598-024-52203-3)
Supplement: Supplementary file 1 — Supplementary Figures. [file 41598_2024_52203_MOESM1_ESM.pdf]

Supplementary Figures – Neural Signatures of Emotion Regulation

Jared Rieck, Julia Wrobel, Antonio R. Porras, Kateri McRae, Joshua L. Gowin

Harmonization of Voxel Distributions

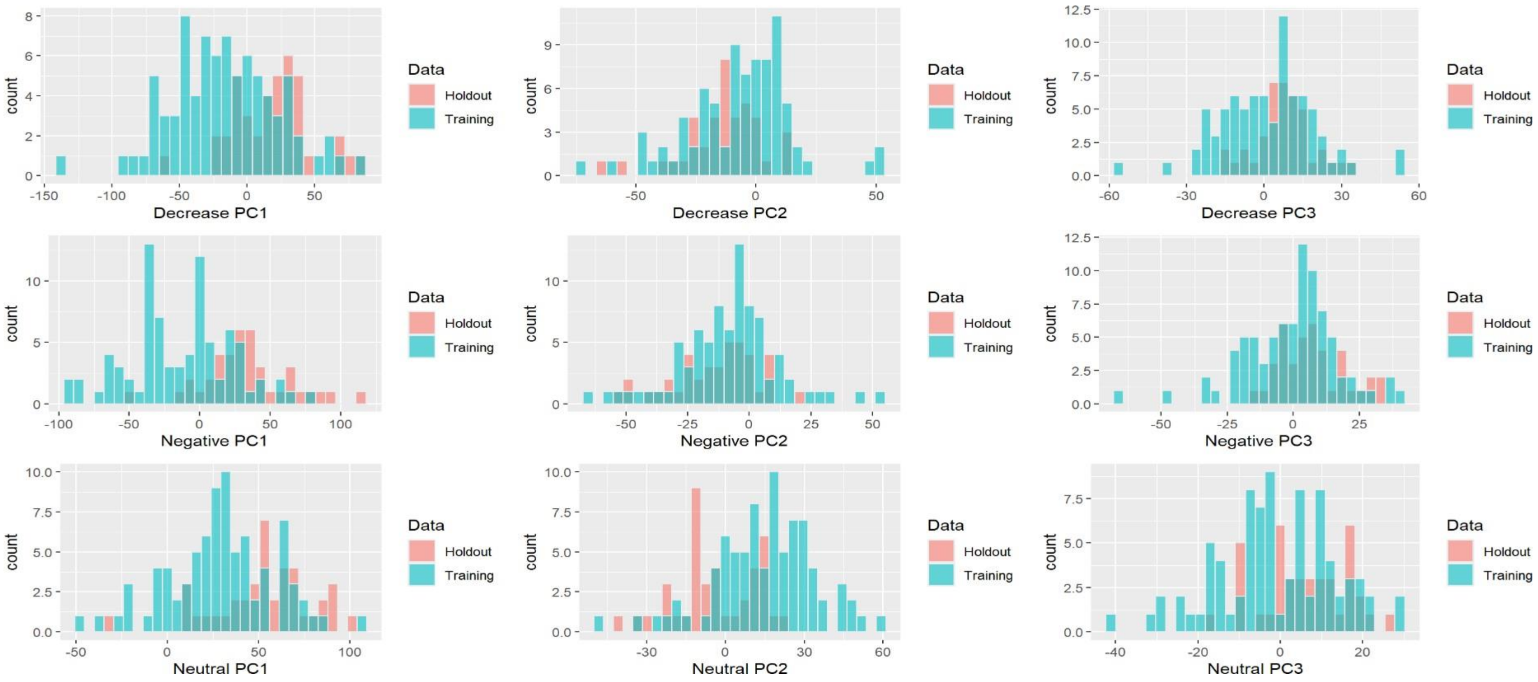

Figure S1: PC Distributions- Pre CovBat

Clear distributional differences are observed in the first three principal components between the training sample (blue) and holdout sample (red) prior to harmonization. The magnitude of distributional differences is modulated by class, with greater similarity observed for the negative classin PC2 and for the decrease class in PC3.

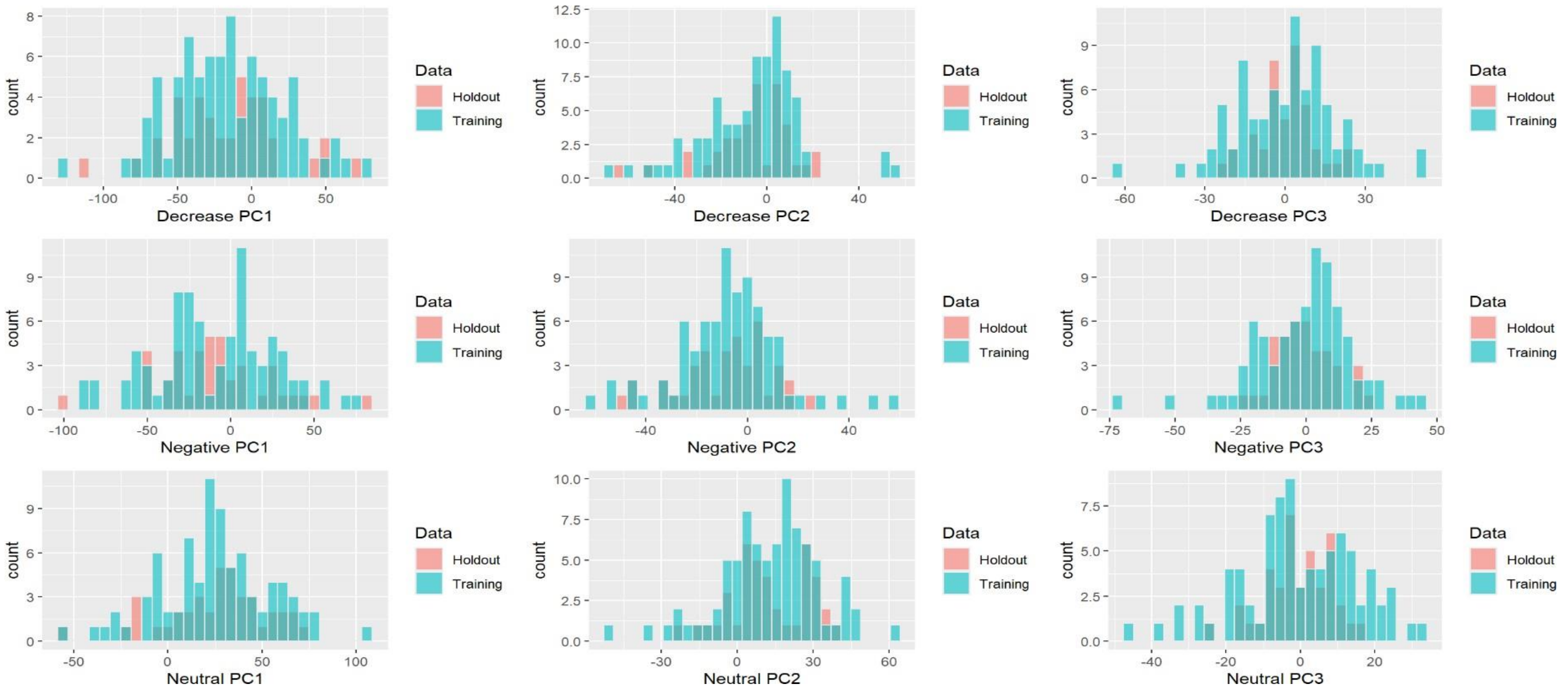

Figure S2: PC Distributions- Post CovBat

After CovBat harmonization, distributional differences between the training and holdout samples in the first three principal components are mitigated, with no clear class modulation effect.
